# Interference Conditions of the Reconsolidation Process in Humans: The Role of Valence and Different Memory Systems

**DOI:** 10.3389/fnhum.2016.00641

**Published:** 2016-12-20

**Authors:** Rodrigo S. Fernández, Luz Bavassi, Laura Kaczer, Cecilia Forcato, María E. Pedreira

**Affiliations:** ^1^Laboratorio de Neurobiología de la Memoria, Departamento de Fisiología y Biología Molecular y Celular, IFIBYNE-CONICET, Facultad de Ciencias Exactas y Naturales, Universidad de Buenos AiresBuenos Aires, Argentina; ^2^Departamento de Física, Facultad de Ciencias Exactas y Naturales, Universidad de Buenos AiresBuenos Aires, Argentina

**Keywords:** reconsolidation, interference, declarative memory, pavlovian conditioning, social threat, boundary conditions

## Abstract

Following the presentation of a reminder, consolidated memories become reactivated followed by a process of re-stabilization, which is referred to as reconsolidation. The most common behavioral tool used to reveal this process is interference produced by new learning shortly after memory reactivation. Memory interference is defined as a decrease in memory retrieval, the effect is generated when new information impairs an acquired memory. In general, the target memory and the interference task used are the same. Here we investigated how different memory systems and/or their valence could produce memory reconsolidation interference. We showed that a reactivated neutral declarative memory could be interfered by new learning of a different neutral declarative memory. Then, we revealed that an aversive implicit memory could be interfered by the presentation of a reminder followed by a threatening social event. Finally, we showed that the reconsolidation of a neutral declarative memory is unaffected by the acquisition of an aversive implicit memory and conversely, this memory remains intact when the neutral declarative memory is used as interference. These results suggest that the interference of memory reconsolidation is effective when two task rely on the same memory system or both evoke negative valence.

## Introduction

Memory interference is defined as a decrease in memory retrieval or performance as a product of new learning. This effect is generated when new information impairs a previously acquired memory. This phenomenon is not only found in laboratory settings, but it is also proposed as the main responsible of forgetting in daily life (Wixted and Ebbesen, 1997; Wixted, 2004; Squire et al., 2015). There are different types of interference that could affect different memory phases (acquisition, consolidation, reconsolidation, or retrieval; Wixted, 2004; Roediger et al., 2007; Besnard et al., 2012; Robertson, 2012; Forcato et al., 2014). Even more, depending on the procedure and timing, the interference could act on memory storage (consolidation), re-storage (reconsolidation) or its retrieval (output). In the first two cases, the observed decrease in memory retention would be irreversible and the stored representation would be impaired (Anderson, 2003; Wixted, 2004; Forcato et al., 2007; Fernández et al., 2016a). On the other hand, a reduced memory retrieval would reflect an inhibitory process or competition between memories. This effect could be reversible by the passage of time or when a recovery protocol is applied (Bouton, 2002, 2004; Anderson, 2003).

Memory storage implies a passage from a fragile state to a stable form, a process called memory consolidation (McGaugh, 2000). However, following the presentation of a memory cue (reminder), consolidated memories become reactivated (labilized), followed by a process of re-stabilization, which is referred to as reconsolidation (Nader et al., 2000; Dudai, 2012; Fernández et al., 2016b). The reconsolidation process is crucial for the modification of existing memories and is the mechanism by which the strength and/or content of consolidated memories are updated (Forcato et al., 2011, 2013, 2014; Inda et al., 2011; de Oliveira Alvares et al., 2012; De Oliveira Alvares et al., 2013; Fukushima et al., 2014). However, it sounds dangerous to put at risk a memory every time it is retrieved. In this context, different reports define boundary conditions for the process (Forcato et al., 2014). Thus, memory features such as strength and age are crucial boundary conditions that limit the beginning of the reconsolidation process (Eisenberg and Dudai, 2004; Milekic et al., 2006; Inda et al., 2011; Dudai, 2012; Forcato et al., 2013).

Reconsolidation is typically revealed by the absence (impairment) of the target memory at testing using pharmacological or behavioral tools (Lee, 2009). In humans, the most common behavioral tool used is memory interference produced by new learning shortly after the reminder presentation (reactivation) of a consolidated memory (Forcato et al., 2014). Several reports showed that the effective time window to impair memory reconsolidation is between 6 and 10 h after memory reactivation (i.e., declarative: Forcato et al., 2007; Kroes et al., 2014; aversive: Nader et al., 2000; Schiller et al., 2010). However, the interference task is usually similar to the target memory (Walker et al., 2003; Forcato et al., 2007; Hupbach et al., 2007; Wichert et al., 2011, 2012; Potts and Shanks, 2012; Fernández et al., 2016a). For example, in human declarative memories, Forcato et al. (2007) used two lists of non-sense syllables, Hupbach et al. (2007) two list of objects and Wichert et al. (2011) two sets of images. Even more, in a motor memory, Walker et al. (2003) used two different finger-tapping tasks. All in all, these results support the idea of a re-storage impairment, and consequently the loss of the original information.

In spite of the profuse research on memory reconsolidation, the conditions under which the interference of this process occurs was scarcely explored. In general, the consolidated memory and the newly acquired one (interference task) share several common elements such as: contextual cues, the task itself, valence, and memory systems (declarative/implicit). Therefore, it remains an open question whether interference between two memories requires an exact match between the tasks to produce memory impairment. This question gains relevance from a neurobiological perspective. Retrieval reactivates the same neural pathways active during initial learning (Shimamura, 2014). This concept implies a competition for shared resources between the reactivated and new memory. Then, we can expect that the reconsolidation of a target memory is disrupted by interference tasks that rely on the same memory system. Moreover, the allocation and synaptic tagging hypothesis (Silva et al., 2009; Rogerson et al., 2014) showed that if a new information shortly follows a learning episode, the very same recently active circuits will be recruited to represent the new memory, producing memory interference. In this background, interference should be effective when the new task can re-use the same neural-substrates as the target memory. The aim of the present study was to investigate the conditions under which the interference of memory reconsolidation occurs. We analyzed when interference occurred across non-identical tasks that use the same vs. different memory systems. With this aim, we used four different learning protocols, which generated two different neutral declarative memories and two implicit aversive memories. In Experiments 1 and 2 we matched memory system and emotional value of the tasks (neutral declarative or implicit negative memories, respectively). In Experiments 3 and 4 we used non identical memory systems and valence, using either a neutral declarative memory or an implicit aversive memory as target or interference memory. Our findings suggest that the reconsolidation of a target memory is disrupted by interference tasks that rely on the same memory system

## Materials and Methods

### Participants

A total of 301 undergraduate and graduate students (170 females and 131 males) from Buenos Aires University (Argentina) mean age of 24±2, participated in the current study. Other 19 subjects were excluded from the analysis based on each protocol inclusion criterion. Prior to the experiments, participants provided a written informed consent that was approved by the Ethics Committee of the Review Board of the Sociedad Argentina de Investigación Clínica. Subjects were randomly assigned to one group.

### Basic Experimental Procedure

**FIGURE 1:**
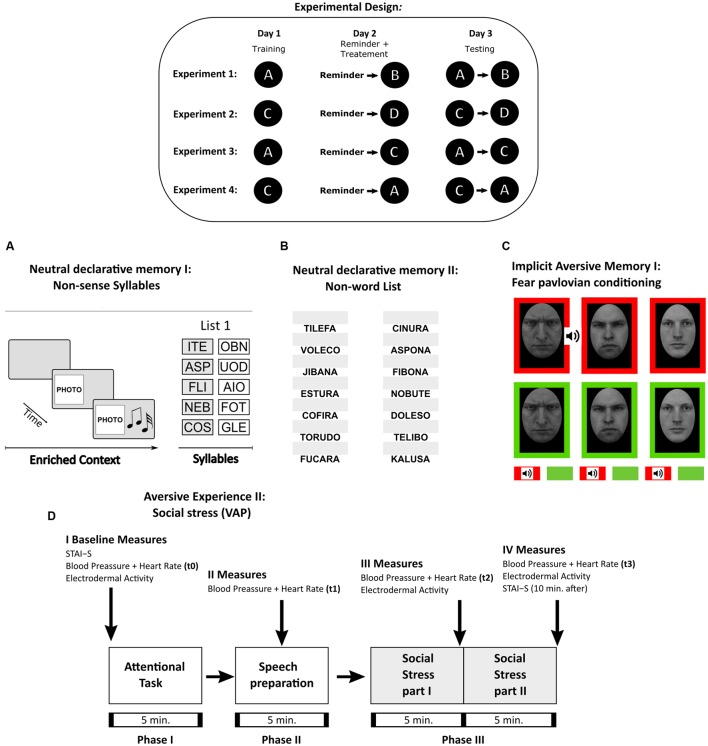
**Experimental Procedures.** Table: Experimental design and the protocols used in Experiments 1–4. Each letter corresponds to the memory protocol used below. **(A)** Neutral declarative memory I (List 1). A trial consisted of the context period, i.e., a specific combination of a light (color illumination of the room), image (a picture) and sound (music), and by a syllable period, i.e., after the stimulus presentation, the five pairs of cue-response syllables (List 1 as shown) were presented successively 10 times. Testing session consisted in the context formation and two cue recall trials. **(B)** Neutral declarative memory II (List NW). Consisted of the presentation of the first syllable of the non-word (left side of the computer screen) and a white box (right side of the computer screen) where subjects completed the entire non-word. In a trial, the 14 non-words were presented. Training consisted in nine trials and testing in two trials. **(C)** Implicit aversive memory (fear pavlovian conditioning). Three male images were used a CS. The stimuli were presented in two different block-contexts, which had different reinforcement probabilities. In the threatening context (red background), the US (a tone) was associated with one of the male angry images (CS1-T) but never with the other two stimuli (CS2-T or CS3-T). The safe context (green background) consisted in the blocks in which the stimuli were never reinforced (CS1-S, CS2-S, and CS3-S). A trial consisted in a CS presentation within each block-context. Each stimulus was presented five times during acquisition and extinction. **(D)** Social threatening event (VAP). Schematic diagram showing timing of the tasks and the different measures obtained: Subjective Rating (STAI), cardiovascular activity (Blood Pressure and Heart Rate), and electrodermal activity (SCL). Social Stress part I refers to the speech in front of an auditory (VAP. Social Stress part II stands for the arithmetic task and was conducted as above. The arrows stand for the time when the measures were taken.

The basic experimental design in all cases included a target memory and an interference task. During day 1, participants were trained with the target memory (training, **Figure 1** inset). The following day (day 2) consisted of a reactivation session of the target memory followed by an interference task. Finally, during day 3 a testing session of both memories was performed. To determine whether the interference specifically affected the target memory reconsolidation we compared the following experimental groups: a Reminder+Interference group (i.e., the reminder is intended to reactivate the target memory and received the interference task), a Reminder group (i.e., reactivation with no interference), noReminder+Interference group (i.e., no memory reactivation) and a Control group (i.e., only acquired the interference task on day 2). Importantly, a 5 min interval was used between target memory reactivation and the interference task, based on previous works of memory reconsolidation in humans that demonstrated it requires at least 6 h for this process to be completed (Forcato et al., 2007; Kroes et al., 2014).

We used four different memory paradigms, either as target memories or interference tasks (**Figure 1** inset). In particular, two were neutral declarative tasks: the non-sense syllables protocol (used in Experiments 1, 3, and 4) and the non-words list (used in Experiment 1). The other two protocols included an emotionally negative component: a fear Pavlovian conditioning (used in Experiments 2, 3, and 4) and a social threatening event (VAP) (used in Experiment 2).

## Experiment 1: Interference of A Neutral Declarative Memory

To determine if the memory system is required to be the same in both the target memory and the interference task we performed Experiment 1. In this experiment, the interference of memory reconsolidation was evaluated using two different neutral declarative memory tasks: the List 1 (target memory) and the List NW (interference task).

**FIGURE 2:**
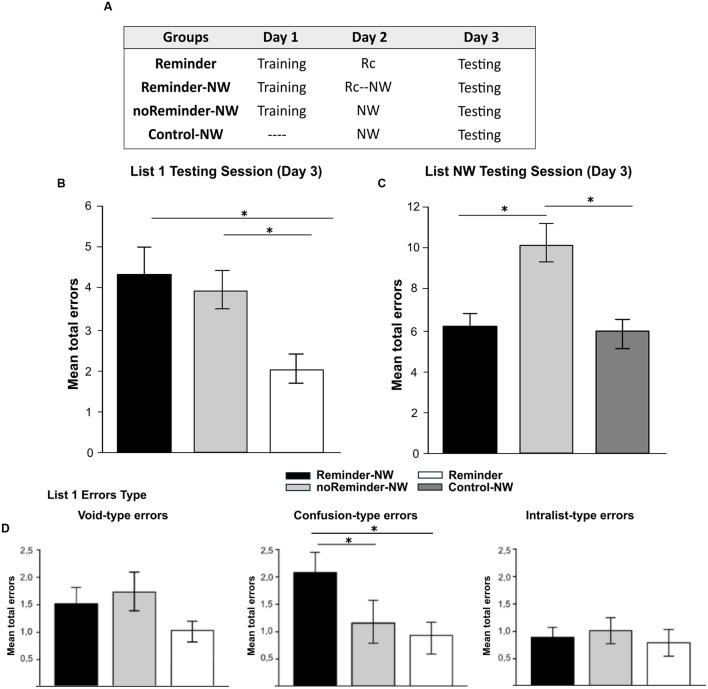
**Experiment 1.** The reconsolidation of a neutral declarative memory (List1) could be interfered by the presentation of a reminder followed by the acquisition of a memory with similar memory system and/or valence on Day 2 (List NW). **(A)** Experimental protocol. A 3 day experiment. **(B)** Target memory testing session (List 1). **(C)** Interference task testing session (List NW). **(D) Error type.** Mean number of total errors ±SEM on Day 3, ^∗^ *p* < 0,05, NW refers to List NW training. Abbreviations as above.

A total of 52 participants (25±2,8 years old) were randomly assigned to four groups, depicted in **Figure 2A** (*n* = 13 per group): Reminder, Reminder-NW, noReminder-NW, and Control-NW groups.

### Target Memory: Non-sense Syllables Protocol (Neutral Declarative Memory)

#### Training Session (Day 1)

Participants learned a list of five pairs of non-sense syllables (List 1) in an enriched specific context (image, colored light and music; for details see Forcato et al., 2007). Each pair was formed by a cue syllable associated with a response syllable (**Figure 1A**). In the first training trial, the List 1 was shown, and in the successive trials subjects were required to write down the corresponding response-syllable for each cue-syllable presented. During the training session, the list was presented in 10 trials. The Training session is reported as the mean number of errors per training trial.

#### Reminder (Day 2)

The reminder was formed by the specific context and a cue-syllable immediately followed by an interruption message without any opportunity to complete the target (Forcato et al., 2009).

#### Testing Session (Day 3)

Consisted of two testing trials. Errors were classified as: Void-type errors (blank responses), associated with memory persistence (Forcato et al., 2013), Confusion-type errors (writing a non-existent response syllable), associated with memory precision (Forcato et al., 2011), and intralist-type errors (writing response syllables for a different cue syllable). Only the subjects that achieved at least 65% of correct responses during the last four training trials were included.

### Interference Task: Non-words Protocol (Neutral Declarative Memory II)

#### Training Session (Day 2)

After the reminder presentation, subjects learned a list of 14 non-words (List NW, **Figure 1B**) with a constant background image in the PC monitor (a forest landscape). The non-words were taken from a rioplatense-Spanish neuropsychological test (Rugg, 1984; Ferreres et al., 2003). In the first training trial the entire List NW was shown. In the successive trials, the first syllable of the non-word appeared on the left side of the screen (i.e., “JI”) and an empty dialog box (a blank rectangle) was shown on the right side, in which participants had to type the entire non-word (i.e., “JIBANA”, **Figure 1D**). A trial ended when the entire list of non-words was presented. Subjects had 6 s to respond and when they answered correctly the word remained in black color for 4 s. Otherwise, the correct response appeared in red color for 4 s. Training session consisted of nine trials.

It is important to highlight that in spite of both tasks (List 1 as target and List NW as interference task) consisted in verbal material (six letters split into two syllables or an entire non-word), they differed in their associative nature (associative vs. non-associative) and context in which it occurred (enriched vs. simple).

#### Testing Session (Day 3):

Participants were tested with two presentations of the non-words list using the same procedure as mentioned above. Only the subjects that achieved at least 65% of correct responses during the last three training trials on Day 2 were included.

#### Amnesic Effect (Retrieval vs. Reconsolidation Impairment)

Considering that memories are integrated into complex associative networks, we used the retrieval-induced forgetting effect as an indirect method to reveal the amnesic effect of the interference task on the target memory reconsolidation (List 1; Anderson et al., 1994). This effect implies that the retrieval of a target memory could temporary block the retrieval of related memories. Because memories share elements (room, experimenter, etc.), it is expected that they interact during retrieval. Thus, if the List 1 memory is intact, its retrieval temporally interferes with the subsequent retrieval of a related memory (Retrieval-Induced Forgetting –RIF-, high number of errors for the interference task at testing). Otherwise, if List 1 is impaired (i.e., memory reconsolidation was interfered), its retrieval does not interfere with the interference task retrieval (no-RIF, few errors for the interference task testing (Forcato et al., 2007).

#### Statistical Analysis

The Training session of List 1 and List NW are reported as the mean number of errors per training trial and was analyzed with repeated-measures ANOVA. The Testing session of both declarative memories were first analyzed with one-way ANOVA and followed by *post hoc* comparisons (FISHER, α = 0,05). We analyzed the types of errors of List 1 in the same manner as testing session.

### Results

#### Neutral Declarative Memory Task (List 1 and List NW)

There were no significant differences between the groups at List 1 training [Repeated-measures ANOVA, List 1 *F*(2,33) = 0,694, *p* > 0,05] as well no group by trial interaction [List 1, *F*(16,264) = 0,399, *p* > 0,05, **Supplementary Figure S1**]. The analysis of List 1 performance at testing (Day 3) showed that the Reminder-NW and the noReminder-NW groups made significantly more errors compared with the Reminder group [**Figure 2B**, one-way ANOVA *F*(2,33) = 4,593, *p* < 0,01, *post hoc* LSD comparison, *p*_reminder-NW_< 0,01 and *p*_noReminder-NW_< 0,05, respectively]. Regarding the error type, the Reminder-NW group committed more Confusion-type errors than the other groups [**Figure 2D**, *F*(2,33) = 5,877, *p* < 0,005, *post hoc* LSD comparison, *p*_noReminder-NW_< 0,05 and *p*_Reminder_< 0,005]. No differences were found for the Void-type and Intralist-type error (*p*_all_> 0,05). Thus, the groups that received the interference List NW (Reminder-NW and noReminder-NW groups) had an increased number of errors independently of receiving the reminder on Day 2. To disentangle if List 1 performance at testing was a result of an impairment effect on memory reconsolidation or retrieval interference, we analyzed the List NW interference task performance (see *amnesic effect* in Section “Interference Task: Non-words Protocol (Neutral Declarative Memory II)”]. Our hypothesis (Forcato et al., 2007) states that when Retrieval-Induced Forgetting (RIF) is revealed (i.e., a high number of errors for List NW at testing), this implies that the target memory remains intact. However, if the target memory is impaired, there should be no-RIF effect (i.e., low number of errors for List NW.

**Figure 2C** shows a similar number of errors at List NW testing in the Reminder-NW and the Control-NW groups, revealing the absence of RIF [Day 3; one-way ANOVA *F*(2,33) = 4,90, *p* < 0,01, *post hoc* LSD comparison *p*_Reminder-NWvs.Control-NW_ *p* > 0,05]. In contrast, the noReminder-NW group committed significantly more errors than the other groups indicating the presence of RIF (*p*_Reminder-NW_ < 0,01 and *p*_Control-NW_< 0,05, respectively). Thus, the acquisition of a different neutral declarative memory (List NW) after the reminder presentation (Reminder-NW group) impaired the reconsolidation of the target neutral declarative memory (List 1). Additionally, the List NW training without memory reactivation by the reminder presentation (noReminder-NW group) only inhibited the retrieval of the target memory (List 1).

These results highlight the importance of shared memory systems in order to impair memory reconsolidation by new learning after the reminder presentation.

## Experiment 2: Interference of An Aversive Implicit Memory

**FIGURE 3:**
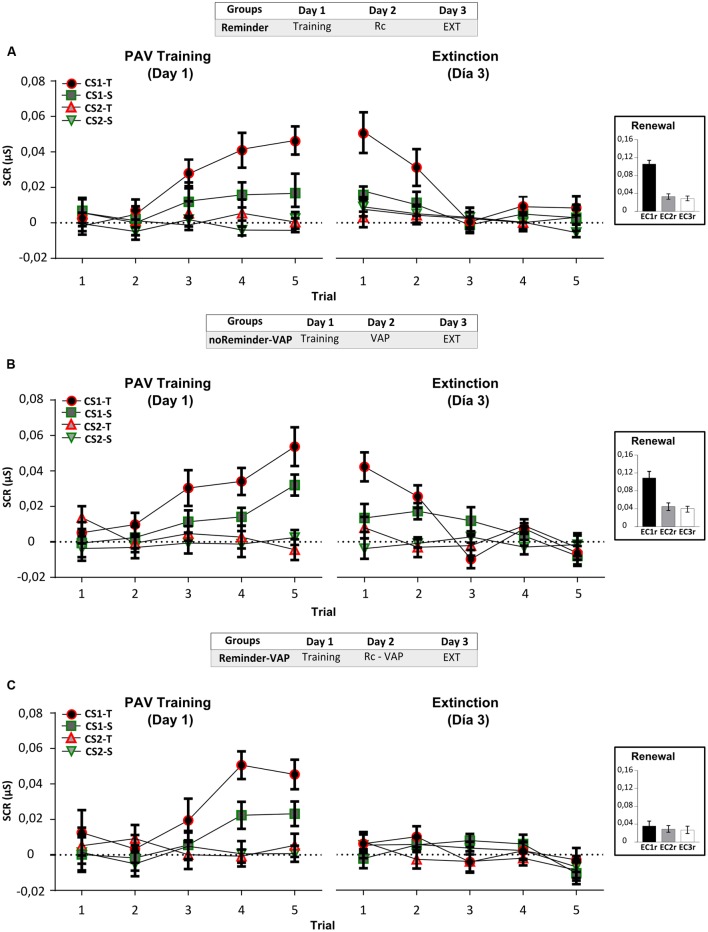
**Experiment 2.** The reconsolidation of an implicit aversive memory (pavlovian conditioning) could be interfered by a social threatening event after its reactivation on Day 2. **(A)** Reminder group. **(B)** noReminder-VAP group. **(C)** Reminder-VAP group. Mean SCR (μS) ±SEM. Left panel: Fear conditioning training (PAV) on Day 1. Right panel: Extinction training on Day 3 (EXT). Right inset: Renewal test after extinction training. VAP refers to the social threatening event.

To study how two similar emotionally negative memories or experiences could produce the interference of memory reconsolidation, we performed Experiment 2. We used an implicit aversive memory protocol (target memory, Fear Pavlovian conditioning) and a social threatening event (interference task, Virtual-Auditory Protocol; Fernández et al., 2015). A total of 52 participants (23±2,5 years old) were randomly assigned to three groups (**Figure 3**) (*n* = 17 per group): Reminder, noReminder-VAP and Reminder-VAP groups.

### Target Memory: Pavlovian Fear Conditioning Protocol (Implicit Aversive Memory)

Pavlovian fear conditioning involves the process by which the representation of two events (stimulus) become associated and then, one of them (CS) is capable of predicting the occurrence of the other (US; Rescorla, 1988; Pavlov and Anrep, 2003). Importantly, fear responses are context-modulated. For example, a tiger represents a real threat in the jungle but not in the zoo (Holland, 1992; Bouton, 2002; Maren et al., 2013). Here we aim to highlight this contextual aspect of fear using a new Pavlovian conditioning which generates a fearful association between cues (CS–US) modulated by two different contexts (threatening and safe contexts, see below).

In order to strengthen the fear association during training, we used fear relevant stimuli as CS (Seligman, 1971; Öhman and Mineka, 2001). The pictures were taken from the *Karolinska Directed Emotional Faces* (KDEF) database (Lundqvist et al., 1998). Three different male faces (CS1, CS2, and CS3, **Figure 1B**),) were presented in the center of a black screen (slides of 9,5 cm × 7 cm. Two of them (CS1 and CS2) expressed anger in their faces and the other one (CS3) was neutral (no emotion).

Each CS was presented in two different block-contexts, with different reinforcement probabilities. In the threatening context, the three CS’s appeared in a red background and the US was presented in 55% of trials, only with one of the CSs (hereafter CS1-T) but never with the other two images (CS2-T or CS3-T). The safe context consisted of the blocks in which the same CSs were presented in a green background and were never reinforced (hereafter CS1-S, CS2-S, and CS3-S). Therefore, only one CS was reinforced in only one context (CS1 in the threatening context: CS1-T). An auditory stimulus (tone) with duration of 1,5 s delivered through stereo headphones served as US. All the CS were presented for 6 s and the US appeared 1,5 s before CS offset. The interval between stimuli varied between 8 s, 10 s, and 12 s. The tone was generated by a TG/WN Tone-Noise Generator (Psychlab), digitally controlled (range 30 to 2000 hz) with a mean of 96 db±4 db. The US was adjusted for each subject to be “unpleasant but not painful” (100 db was the maximum allowed for any subject).

#### Training Session (Day 1)

On day 1, subjects were informed that one of the slides specifically in one context would be followed by an unpleasant tone (US) in most of the cases. They were also instructed to pay attention to the color-contexts and to predict the US arrival (US expectancy) with an external keypad (YES/NO buttons) during the expectancy period of each CS (YES/NO boxes appeared on the screen for 3 s). Stimuli were presented during three block-contexts (threatening/safe). Each CS appeared five times for 6 s in each context (threatening/safe) with the exception of CS1-T (the reinforced CS in the threatening context) which was presented 11 times (55% of reinforcement, **Figure 1B**). The order of block-contexts and CS presentation was pseudo-randomized. Finally, assignment of the aversive slides (CS1 and CS2) as the reinforced stimulus in the threatening context was counterbalanced across subjects.

Fear acquisition was measured by the skin conductance response (SCR). The input device (*Psychlab Precision Contact Instruments*, www.psychlab.com) has a sine excitation voltage (±0,5 V) of 50 Hz derived from the main frequency. The device was connected to two Ag/AgCl electrodes of 20 mm × 16 mm located in the intermediate phalanges of the index and middle fingers of the non-dominant hand. SCR produced by each CS was measured by taking the average baseline to the first peak within the 0,5-6 s window following stimulus onset. For the CS1-T, only the non-reinforced trials were analyzed. A minimum response criterion of 0,002 micro Siemens (μS) was used and all the other responses were scored as zero (Kindt et al., 2009; Boucsein, 2012). The raw SCR scores were square-root transformed to normalize distributions. Since fear-relevant stimulus usually have stronger SCR amplitudes and generalizes faster (Seligman, 1971; Öhman and Soares, 1998; Lissek et al., 2005), we used the mean differential SCR between each fear-relevant CS (CS1 and CS2) and the neutral one (CS3; Schiller et al., 2010; Agren et al., 2012) in each context (i.e., CS1-T–CS3-T), given a total of 4 stimuli analyzed: CS1-T, CS1-S, CS2-T, and CS2-S. Data was analyzed with Matlab (Mathworks Inc. Sherborn, MA, USA) and Ledalab (Benedek and Kaernbach, 2010). Only subjects who showed differential fear responding (CS1-T SCR amplitude > others CS’s) during acquisition were considered for analysis.

#### US Expectancy

During CS presentation (in each context) subjects were instructed to press YES or NO button (expectancy keys of the external keypad) when the YES/NO boxes appeared in the inferior section of the screen. The subject has to press YES if he thinks that the US will follow that CS and NO if otherwise. US expectancy was analyzed using the mean of errors committed in the US prediction for each context (threatening/safe) and trial.

#### Stimuli Aversiveness

After the end of training (Day 1) and extinction sessions (Day 3), subjects were instructed to rank in a 1 to 10 scale how aversive or unpleasant was each CS (CS1, CS2, and CS3 images) and the US (tone).

#### Pavlovian Reminder (Day 2)

We developed a reminder based on previous findings with the non-sense syllables protocol (Forcato et al., 2009) and taking into account the fact that the US omission (a negative prediction error) is the most typical kind of reminder (Kindt et al., 2009; Schiller et al., 2010; Fernández et al., 2016b). Thus, the reminder consisted of an unreinforced presentation of the CS1-T (i.e., a negative prediction error). First, the CS1-T (the CS associated with the US in the threatening context) was presented and then the YES/NO boxes (US expectancy) were shown for 3 s. Finally, the CS1-T disappeared (without US presentation) followed by an interruption message announcing that the experiment was finished.

#### Testing Session: Extinction (Day 2 or 3)

Memory extinction refers to the unreinforced presentation of the stimulus previously associated with the US in each context (CS1-T; Bouton, 2004). As a consequence, the intensity of the fear response gradually decreases with each trial. Memory extinction implies the formation of a new inhibitory memory, which competes with the original one and allows the use of recovery protocols (Bouton, 1993, 2002, 2004). During the extinction session, participants put the headphones on and they were told that the session would be similar to day 1. The presentation, duration, number and intervals of the stimuli was the same as in Day 1 and the subjects had to predict US arrival with the external keypad (US expectancy). During extinction training, no US (tone) was presented with any stimulus or context.

#### Renewal (Recovery Protocol, Day 2 or 3)

Following extinction training, we used a renewal protocol to assess memory recovery. The rationale for this procedure is that recovery protocols (reinstatement, spontaneous recovery, renewal) are a powerful tool to differentiate reconsolidation impairment from extinction memory (Kindt et al., 2009; Schiller et al., 2010). That is, if the reconsolidation process is impaired, the recovery protocol is unable to rescue the original information. On the other hand, because of the inhibitory nature of extinction, the original fear memory can be recovered after this experimental manipulation. Here we used *renewal* as a recovery protocol, which consists of the presentation of the stimuli in a different or new context (Bouton, 1993). In our experiments, after extinction was completed, the CS1, CS2, and CS3 were presented only once with a black background creating a new/different context (hereafter CS1r, CS2r, and CS3r). The duration and inter-trial intervals were similar to the other sessions. Here, subjects also had to predict US arrival (US expectancy) and were asked to evaluate the aversiveness of all the stimuli in a 1 to 10 scale after the session had finished.

#### Statistical Analysis

##### (a) Electrodermal activity (SCR)

Skin conductance response amplitudes of the difference between the fear relevant CS (CS1 and C2) and the neutral one (CS3) in each context (threatening/safe) was analyzed by means of a mixed analyses of variance (ANOVA) for Repeated-measures with Group as between-subjects factor and stimulus (CS1-T, CS1-S, CS2-T, and CS2-S) and trial (trials 1-5 of acquisition and extinction) as within-subjects factors. The differential stimulus response (CS1-T vs. others CS’s) was analyzed over time during acquisition (first trial vs last trial), retention (last trial of acquisition vs. first trial of extinction) and extinction (first trial vs. last trial). When the interaction was significant, simple effects were performed followed by LSD comparisons when appropriate. The renewal test (CS1r vs. CS2r vs CS3r) was analyzed using a two-factor ANOVA (group × stimulus) and followed by LSD comparisons.

##### (b) US expectancy

US expectancy was analyzed using the mean of errors committed in the US prediction for each context (threatening/safe) and trial. During acquisition, an error was considered when participants predicted the US with the incorrect CS or did not predict the US with the correct one. Then, during the extinction session, since no US was presented, any positive response was considered an error. The absence of response was not analyzed. US expectancy errors were analyzed by means of a mixed repeated-measures ANOVA similar to the electrodermal activity (SCR): Group as between-subjects factor and two within-subjects factors: Context (threatening/safe) and trial (acquisition and extinction). Since during the renewal test it was not possible to obtain an error (since there is no previous rule to predict the US), subjects’ responses were reported as percentage of YES/NO responses to each stimulus (CS1r, CS2r, and CS3r).

##### (c) Stimuli aversiveness

The evaluative component of Pavlovian conditioning was analyzed similar to the US expectancy with a mixed repeated-measures ANOVA: Group as between-subjects factor and two within-subjects’ factors: stimulus (CS1, CS2, CS3, and US) and day (day 1 or day 2 vs. day 3).

### Interference Task: Social Threatening Event: Virtual-Auditory Panel (VAP)

The virtual-auditory panel (VAP) protocol (see detailed description in Fernández et al., 2015) is an adaptation of the Trier Social Stress Test (TSST) protocol. It consisted of three phases (**Figure 1C**). *Phase 1* was an undemanding attentional task, in which 16 landscape images were shown and participants were asked to rate the images according to their likes. In *Phase 2*, participants had to prepare a speech to advertise themselves as the best candidate for a professional position; this phase lasted 5 min. Finally, in *Phase 3*, the experimenter explained to the participants that a hospital committee was following the presentation online using a webcam. As in the TSST protocol (Kirschbaum et al., 1993), after the presentation, participants had to perform an arithmetic task. The experimenter used a pre-recorded ambient sound (different office sounds such as engines, keys, chairs, etc.) as background and a pitch modifier provided with three different voices (virtual panel) that simulated a hospital committee.

#### Measurements

Baseline measurements for the State Trait Anxiety Inventory (STAI, Spielberger et al., 1970), blood pressure, and heart rate were taken before *Phase 1*. Blood pressure and heart rate were measured at four different time points: t0 (before *Phase 1*), t1 (after *Phase 2*), t2 (after the speech presentation), and t3 (after the arithmetic task; **Figure 1C**). Skin conductance level (SCL) was recorded during the entire experiment; we defined the SCL baseline level as the continuous measure during *Phase 1*. Blood pressure, heart rate and the STAI were measured for the last time at the end of *Phase 3*.

#### Statistical Analysis

##### (a) STAI

The STAI is reported as the mean score difference in each participant at the end of *Phase 3* and before the attentional task (*Phase 1*). Data were analyzed using one-way analysis of variance (ANOVA).

##### (b) Blood pressure and heart rate

A mean cardiovascular value (t0, t1, t2, t3) was reported (mm/HG, BPM). Data were analyzed using Repeated-measures ANOVA (Group × Time). When sphericity was not accomplished, Greenhouse-Geisser correction was applied.

##### (c) Electrodermal activity (SCL)

It is reported as the mean SCL (μS) in each participant during the baseline attentional task (*Phase 1*) and during stress induction (*Phase 3*). The use of the mean SCL is supported by the stationary time series of the signal and the low variability between points (Fernández et al., 2015). Data were analyzed using repeated-measures ANOVA (Group × Time).

### Results

#### Implicit Aversive Memory Task (Fear Pavlovian Conditioning)

##### (a) Electrodermal activity (SCR)

Acquisition on Day 1, measured by the SCR, showed fear conditioning in all groups by the significant increase in CS1-T SCR levels from trials 1 to 5 [**Figure 3**, left panel: Mixed repeated-measures ANOVA, Stimulus × Trial Interaction: *F*(9,405) = 3,948, *p* < 0,001, simple effects *p*_CS1-T_< 0,001; Stimulus × Group Interaction: *F*(6,135) = 0,530, *p* > 0,05], and in relation to the non-reinforced stimulus (Trial 5, simple effects *p*_CS1-Tvs.all_ < 0,05). Interestingly, on Day 3 only the group which received the Pavlovian reminder followed by the social threatening event (Reminder-VAP group) significantly decreased CS1-T electrodermal activity (**Figure 3**, right panel). An independent Repeated-measures ANOVA revealed the absence of memory retention from the last trial of acquisition to the first trial of extinction [**Figure 3C**, Reminder-VAP group, *F*(1,15) = 10,096, *p* < 0,005] and the same profile was found when comparing its SCR amplitude to the other stimulus [Factor Stimulus: *F*(3,45) = 0,938, *p* > 0,05). Furthermore, SCR levels during extinction in this group remained similar from trials 1 to 5 (*p* > 0,05). However, the Reminder and noReminder-VAP groups, showed comparable SCR levels to the Pavlovian training on Day 1 (Trials 5-1, *p* > 0,05). In both groups, a significant decrease in SCR was observed during extinction training from trials 1 to 5 (Trial: simple effects *p*_CS1-T_ < 0,001). Regarding the renewal test, a Two-way ANOVA revealed the recovery of fear (SCR) for CS1r but not CS2r and CS3, only in the Reminder and noReminder-VAP groups [**Figures 3A,B**, inset; Group × Stimulus interaction: *F*(4,135) = 2,642, *p* < 0,05; Stimulus Factor: *F*(2,135) = 10,30, *p* < 0,01; *post hoc* LSD comparisons *p*_CS1r-CS2r_ *p* < 0,001, *p*_CS1r-CS3r_ *p* < 0,001 y *p*_CS2r-CS3r_> 0,05]. In addition, in the Reminder-VAP group no differences were found between stimuli (**Figure 3C**, LSD: *p*_CS1r-CS2r_ *p* > 0,001, *p*_CS1r-CS3r_ *p* > 0,001 and *p*_CS2r-CS3r_ > 0,05) suggesting that memory reactivation before the threatening event blocked the return of fear by impairing its reconsolidation.

##### (b) US expectancy

All groups accurately predicted the US with the CS1-T presentation during acquisition on Day 1 [Figure S2, repeated-measures ANOVA, Context × Trial Interaction: *F*(9,1098) = 11,720, *p* < 0,001, Threatening context: Trials 1-2 *p*_1-2_< 0,001] and predicted it during the first trial of extinction on Day 3 (Threatening context p_5-1_< 0,001; simple effects *p*_contexts_ *p* < 0,001). Moreover, during trials 1-5 of extinction training participants learned that the US was not delivered with any stimulus (Trials 1-2 *p*_1-2_< 0,01; Trials 4-5 p_4-5_> 0,05). Regarding the renewal test, nearly half of the subjects of each group predicted the US when the CS1r was presented but not with the CS2r and CS3r (**Supplementary Table S1**) suggesting that the participants had not the proper information to make a precise prediction about US occurrence.

##### (c) Stimuli aversiveness

Ratings about stimulus aversiveness were comparable across groups and stimulus between Day 2 and Day 3 [**Supplementary Table S2**; Group × Stimulus interaction *F*(4,90) = 0,552, *p* > 0,05]. We found as expected that: **(1)** the fearful CS (CS1 and CS2) were rated as more unpleasant than the neutral CS3 [Stimulus: *F*(2,90) = 306,264, <0,001, *post hoc* LSD comparisons *p*_CS1-CS3_< 0,01, *p*_CS2-CS3=_ < 0,01, and *p*_CS1-CS2_> 0,05] and **(2)** a generalized decrease in subjective ratings between days [Trial: *F*(1,45) = 4,48, *p* < 0,05].

#### Aversive experience task (VAP)

All groups which experienced the social threatening event (Reminder-VAP and noReminder-VAP) exhibited a significant increase in sympathetic nervous system activity and cognitive stress following its administration (**Supplementary Table S3**). The VAP protocol induced an increment in cardiovascular and electrodermal activity without any differences between groups. Both VAP groups also showed similar STAI scores, which suggests an increase in subjective stress and overall a successful sympathetic activation (**Supplementary Table S3**).

From these results, we showed that a social threatening event (VAP) after fear memory reactivation could impair the memory reconsolidation. On the other hand, the US expectancy and reported aversiveness of stimuli (**Supplementary Figure S2**; **Supplementary Tables S1** and **S2**) were not affected in any group, suggesting, as in other reports (Soeter and Kindt, 2010), that the expectancy and implicit component of this memory could be independent. These indicate that, in order to be effective, the interference task used to impair memory reconsolidation requires sharing memory system (declarative/implicit) and/or valence (neutral/aversive).

## Experiment 3 Interference of A Neutral Declarative Memory II

The goal of Experiment 3 was to determine whether the reconsolidation of a neutral declarative memory (List 1) could be interfered by an aversive implicit memory (fear Pavlovian conditioning).

**FIGURE 4:**
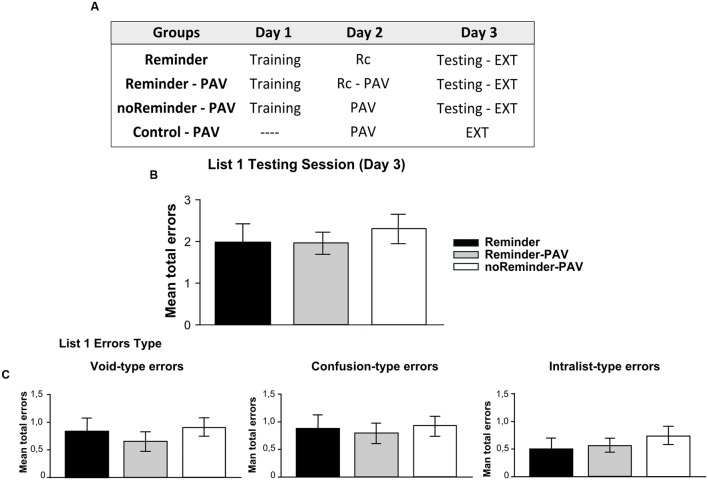
**Experiment 3 (List 1).** A neutral declarative memory is not affected or interfered by the acquisition of an implicit aversive memory on Day 2. **(A) Experimental protocol.** A 3 day experiment. **(B)** Target memory (List 1) testing session. **(C) Error type.** Mean number of total errors ±SEM on Day 3. Rc, refers to the presentation of the reminder, PAV to pavlovian conditioning training, and EXT to extinction training.

A total of 51 participants (24±2,1 years old) were randomly assigned to four groups (**Figure 4A**) (*n* = 13 per group): Reminder, Reminder-PAV, noReminder-PAV, and Control-PAV groups.

### Target Memory: Non-sense Syllables Protocol (Neutral Declarative Memory)

We used the same task as in Experiment 1 as target memory.

### Interference Task: Pavlovian Fear Conditioning Protocol (Implicit Aversive Memory)

We used the same procedure as described in Experiment 2 as interference task.

### Results

#### Neutral Declarative Memory Task (List 1)

There were no significant differences between the groups at training of the target memory [Repeated-measures ANOVA, List 1 *F*(2,43) = 0,578, *p* > 0,05]. In addition, there was no group by trial interaction [List 1, *F*(16,344) = 0,571, *p* > 0,05, **Supplementary Figure S1**]. On day 3 (testing session), all groups had a similar performance. We found no differences between groups at testing, irrespectively, of receiving or not the reminder or the Pavlovian learning [**Figure 4B**, One-way ANOVA *F*(2,45) = 0,263, *p* > 0,05]. The analysis of error types revealed also the absence of difference between groups [**Figure 4C**, One-way ANOVA, Void-type errors *F*(2,45) = 0,042, *p* > 0,05; Confusion-type errors *F*(2,45) = 0,052, *p* > 0,05; Intralist-type errors *F*(2,45) = 0,199, *p* > 0,05].

#### Implicit Aversive Memory Task (Fear Pavlovian Conditioning)

**FIGURE 5:**
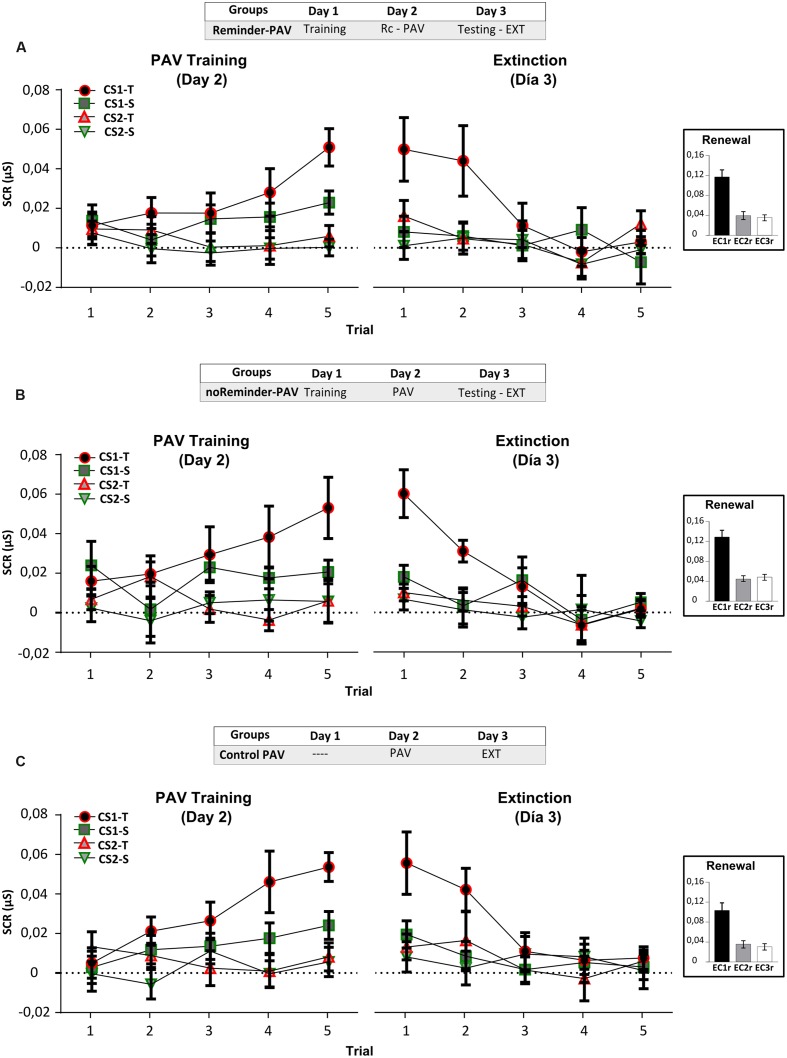
**Experiment 3 (fear pavlovian conditioning).** Learning a fear pavlovian conditioning is not affected by the previous acquisition of a neutral declarative memory (List 1). **(A)** Reminder-PAV group. **(B)** noReminder-PAV group. **(C)** Control-PAV. Mean SCR (μS) ±SEM. Left panel: Fear conditioning training (PAV) on Day 2. Right panel: Extinction training on Day 3 (EXT). Right inset: Renewal test after extinction training.

Analysis of memory acquisition by the SCR showed an equivalent fear learning on Day 2 for all groups (**Figure 5** left panel, Mixed repeated-measures ANOVA Stimulus × Group *F*(6,135) = 0,506, *p* > 0,05), from the first to the last trial of acquisition [trial 1–5; Stimulus × Trial *F*(9,405) = 3,24, *p* < 0,001], specifically for the reinforced CS in the threatening context (CS1-T; simple effects *p*_CS1-T_< 0,01). The absence of differences between the last trial of acquisition on Day 2 to the first extinction trial on Day 3 revealed memory retention for all groups (**Figure 5** right panel; *p*_CS1-T_> 0,05). The increased SCR level for CS1-T in all groups differed from the others stimulus (simple effects, first extinction trial *p*_CS1-T/othersCS_ < 0,005). Finally, SCR significantly decreased in all groups from the first extinction trial to the last one (1–5 trials, simple effects *p*_CS1-T_< 0,001). Moreover, no differences were found between stimulus in the last extinction trial (trial 5, *p*_all_> 0,05) suggesting a successful extinction training. We then analyzed the renewal test in order to unveil this inhibitory memory and recover the fearful response. A Two-way ANOVA revealed a significant increment in SCR levels only for CS1r but not for CS2r and CS3r in all groups [**Figure 5**, inset; Group × Stimulus interaction *F*(4,135) = 0,179, *p* > 0,05; Stimulus factor *F*(2,135) = 11,21, *p* < 0,001, *post hoc* LSD *p*_CS1r-CS2r_ *p* < 0,001, *p*_CS1r-CS3r_ *p* < 0,001 y *p*_CS2r-CS3r_> 0,05]. As a whole, the analysis of the SCR levels for CS1-T demonstrated that this fear Pavlovian memory could be acquired, retained, extinguished, and recovered. US expectancy and the reported aversiveness of stimulus were correctly acquired (Supplementary Results; **Supplementary Figure S2**; **Supplementary Tables S1** and **S2**).

The results from Experiment 3 suggest that a neutral declarative memory (List 1) and an implicit aversive memory (fear Pavlovian conditioning) could be independently acquired and without proactive or retroactive interference. Furthermore, the reconsolidation of the neutral declarative memory (List 1) triggered by the reminder was not affected by the Pavlovian conditioning training.

## Experiment 4: Interference of An Aversive Implicit Memory II

Experiment 4 consisted in a mirrored design of Experiment 3, to evaluate further the possibility of interfering reconsolidation of memories with different system and/or valence. Besides, we wanted to discard any specific characteristics of the neutral declarative memory (List 1) as a target memory or the implicit aversive memory (Pavlovian conditioning) as the interference task (**Figure 7A**).

A total of 68 participants (23±2,2 years old) randomly assigned to four groups were included in Experiment 4 (*n* = 17 per group): Reminder, Reminder-L1, noReminder-L1, and Control L1 groups.

### Target Memory: Pavlovian Fear Conditioning Protocol (Implicit Aversive Memory)

We used the same task as described in previous experiments as target memory.

### Interference Task: Non-sense Syllables Protocol (Neutral Declarative Memory)

We used the same procedure as described in Experiments 1 and 2 as interference task.

### RESULTS

#### Implicit Aversive Memory Task (Pavlovian Fear Conditioning)

**FIGURE 6:**
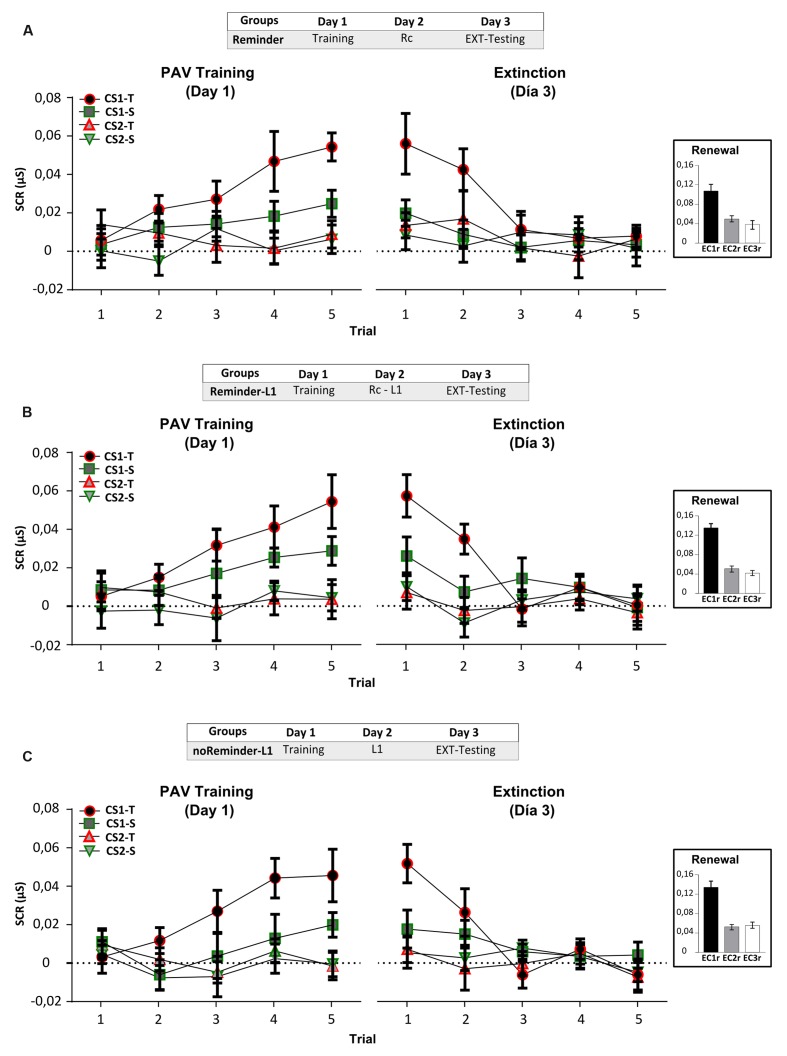
**Experiment 4 (pavlovian conditioning).** An implicit aversive memory is not affected by the acquisition of a neutral declarative memory (List 1) on Day 2. **(A)** Reminder group. **(B)** Reminder-L1 group. **(C)** noReminder-L1 group. Mean SCR (μS) ±SEM. Left panel: Fear conditioning training (PAV) on Day 1. Right panel: Extinction training on Day 3 (EXT). Right inset: Renewal test after extinction training. L1 refers to List 1 training and testing to its evaluation. Abbreviations as above.

On Day 1 we found a successful fear conditioning in all groups (**Figure 6**, left panel), as well as retention – extinction (**Figure 6** right panel and inset; Supplementary Results). The acquisition of the neutral declarative memory (List 1) after the Pavlovian reminder did not affect SCR (implicit component), US expectancy or the reported aversiveness of stimulus (**Supplementary Figure S2**; **Supplementary Tables S1** and **S2**).

#### Neutral Declarative Memory Task (List 1)

**FIGURE 7:**
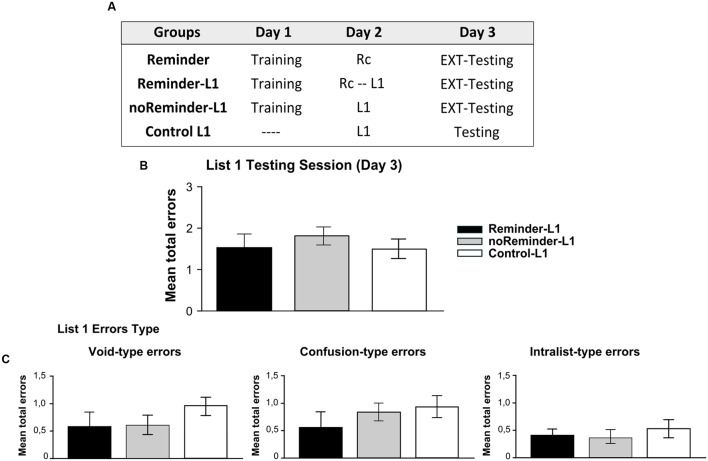
**Experiment 4 (List 1).** Learning a neutral declarative memory is not affected by the previous acquisition of an aversive implicit memory (pavlovian conditioning). **(A)** Experimental protocol. **(B)** Target memory (List 1) testing session. **(C) Error type.** Mean number of total errors ±SEM on Day 3. Abbreviations as above.

A repeated-measures ANOVA indicated no significant differences between the groups for List 1 training on Day 2 [**Supplementary Figure S1**; *F*(2,43) = 0,12, *p* > 0,05] as well as no group by trial interaction [*F*(16,344) = 0,58, *p* > 0,05]. The evaluation of List 1 memory on Day 3 demonstrated that all groups made a similar number of errors (**Figure 7B**, One-Way ANOVA *F*(2,45) = 0,171, *p* > 0,05) irrespectively of its type [**Figure 7C**, Void-type: *F*(2,45) = 0,342, *p* > 0,05; Confusion-type : *F*(2,45) = 0,642, *p* > 0,05; Intralist-type: *F*(2,45) = 0,383, *p* > 0,05]. These findings reveal that the acquisition of a neutral declarative memory (List 1) after a Pavlovian-reminder did not affect the implicit aversive memory, and that this memory is not altered by the previous acquisition of the other one. Taken together, these results replicate those founded in Experiment 3, showing that two independent memory systems (declarative vs implicit) with different valence (neutral vs negative) are not affecting each other’s reconsolidation, retention or acquisition.

## Discussion

The present findings demonstrate that behavioral interference of the reconsolidation process requires a match between memory systems supporting the target and the interfering memory. Typically, the acquisition of a task similar to the target memory after reactivation leads to the interference of memory reconsolidation (Walker et al., 2003; Forcato et al., 2007; Hupbach et al., 2007; Wichert et al., 2011). Here, we first evaluated the effect of an interference task similar (but not identical) to the target memory on the reconsolidation process. We found that the acquisition of a different neutral declarative memory (non-words list) after memory reactivation impairs the reconsolidation of the target neutral declarative memory (non-sense syllables, Experiment 1). Moreover, we showed that an aversive social experience (VAP) impaired the reconsolidation of a reactivated implicit aversive memory (fear Pavlovian conditioning, Experiment 2). Notably, this constitutes the first finding that showed reconsolidation of a Pavlovian fear conditioning in humans could be impaired after its reactivation by a social threatening event. Then, we analyzed memory reconsolidation interference when the tasks used are dissimilar in its system and/or valence. We showed that the acquisition of an implicit aversive memory (fear Pavlovian conditioning) is unable to impair the reconsolidation of a neutral declarative memory after its reactivation (non-sense syllables, Experiment 3) and that this memory does not affect the first one after the reminder presentation (Experiment 4).

A limitation of our experiments is that we did not manipulate one variable (i.e., valence) and maintain constant the other variable (i.e., memory system), in order to disentangle the weight of each factor (performing all the possible interactions). Future experiments have to be performed to characterize these variables, for example, using a list of negative emotional words or stories (declarative negative memory).

In the memory reconsolidation field, the conditions under which behavioral interference occurs were scarcely explored. Animal studies showed that the reconsolidation of a fearful memory could be disrupted after its reactivation followed by a distractor stimuli (air puff) or the exploration of a hole board (Boccia et al., 2005; Crestani et al., 2015). In humans, for example, Schwabe and Wolf (2009) showed that learning a neutral story after reactivation of different autobiographical memories (positive, negative, or neutral), reduced specifically the retention of neutral memories. Additionally, Kredlow and Otto (2015) found that a trauma-related memory could be interfered by a negative story.

Numerous investigations in other memory phases (i.e., acquisition or consolidation) unveiled that the interference between memories could be independent from the type of material used in both tasks (Byrne, 2010). For example, McGeoch and McDonald (1931) reported memory interference between different verbal materials (parts of speech, synonyms, antonyms). Müller and Pilzecker (1970) seminal work showed that a set of non-sense syllables could be either interfered by the acquisition of another set of syllables or a set of images. Beyond the similarity between tasks, it was proposed the task “cognitive-load” as a powerful amnesic agent to the extent that it interrupts the elaborative rehearsal process associated with memory acquisition (Spear, 1973; Wixted, 2004; Dewar et al., 2007). A classic Skaggs’s (1925) study found that the retention of chess positions decreased after solving algebra problems. More recently, Dewar et al. (2007) found the same effect with a word-list when using images, arithmetic problems, a set of clips, or others cognitive-loaded tasks. However, memory systems are not isolated and they might interact. Thus, some authors suggested that memory interference occurs when the acquired memory and the new one activate similar brain structures which overlap and compete for limited neuronal resources (Keisler and Shadmehr, 2010; Cohen and Robertson, 2011; Robertson, 2012). Therefore, different memory systems and/or valences may interfere each other when the representations activated and the neuronal structures involved, overlap and compete. For example, Keisler and Shadmehr (2010) reported interference between motor and declarative memory systems. This could be understood considering that the hippocampus not only plays a major role in declarative memories but it was also found to be associated with the consolidation of motor memories (Shadmehr and Holcomb, 1997; Squire, 2004; Keisler and Shadmehr, 2010). Albeit other processing structures involved in cognitive and executive control, such as the dorsolateral cortex or the hippocampus, may regulate the interaction between memories and modulate the fate of memories and their interference (Anderson, 2003; Costanzi et al., 2009; Kuhl et al., 2010; Cohen and Robertson, 2011; Robertson, 2012).

Our results are in line with these ideas, suggesting that when non-overlapping or partially overlapping memory systems are engaged during memory reconsolidation, memory interference does not occur (Experiments 3 and 4). However, when there is high-overlapping memory systems (re-use of similar pathways) which compete for neuronal resources, memory reconsolidation could be interfered (Experiments 1 and 2). All in all, these findings reveal that in the same way that reconsolidation does not occur every time a memory is retrieved, interference is not produced every single time a new memory is acquired.

## Author Contributions

Conceived and designed the experiments: RF, LK, MP. Performed the experiments: RF. Analyzed the data: RF, LB, CF, MP. Contributed reagents/materials/analysis tools: RF, LK, CF, MP. Wrote the paper: RF, MEP.

## Conflict of Interest Statement

The authors declare that the research was conducted in the absence of any commercial or financial relationships that could be construed as a potential conflict of interest.
