# Supplementary material for: Interference Conditions of the Reconsolidation Process in Humans: The Role of Valence and Different Memory Systems
Source: Front Hum Neurosci. 2016 Dec 20;10:641. doi: 10.3389/fnhum.2016.00641 (PMC5167735; doi:10.3389/fnhum.2016.00641)
Supplement: Supplementary file 1 [file Data_Sheet_1.doc]

**Supplementary Information**

**Interference conditions of the reconsolidation process in humans: interaction between memory systems and valance.**

**Rodrigo S. Fernández^a^, Luz Bavassi^a,b^, Laura Kaczer^a^, Cecilia Forcato^a^&María E. Pedreira^a*^**

**^a^**Laboratorio de Neurobiología de la Memoria, Departamento de Fisiología y Biología Molecular y Celular, IFIBYNE-CONICET, Facultad de Ciencias Exactas y Naturales, Universidad de Buenos Aires, Argentina.

**^b^**Departamento de Física, Facultad de Ciencias Exactas y Naturales, Universidad de Buenos Aires, Argentina.

**Supplementary Results**

**Experiment 1**

**List NW training.**

On day 2, all groups had a similar acquisition during List NW training (Repeated-measures ANOVA, List NW F (2,33) = 0,432 , p > 0,05) as well no group by trial interaction (List NW, F (14,231) = 0,986 p > 0,05, Supplementary Information Figure S1).

**Experiment 3.**

**Implicit aversive memory (fear Pavlovian conditioning).**

*b) US expectancy.* During acquisition on Day 2, subjects in all groups quickly learn to predict the US with the correct CS only in the threatening context (CS1-T, Context x Trial interaction F (9,109) = 12,370, p < 0,001, Threatening context: Trial p_1-2_ < 0,001). The analysis of error between the last acquisition trial and the first extinction trial revealed memory retention (Threatening context p_5-1_< 0,001). Finally, on Day 3 during extinction, all groups no longer predicted the US with any CS or context (Trial p_1-2_< 0,01 and trial p_4-5_ > 0,05). Regarding the renewal test, we found the same patterns of results as in previous Experiment 2 (Table S1).

*c) Stimuli aversiveness.* We found similar results as in Experiment 2 in all groups (Tables S2 and S3,Group x Stimulus Interaction: F (4,90) = 0,104, p > 0,05;Trial Factor: F (1,45) = 16,449, < 0,001; Stimulus Factor: F (2,90) = 265,205, p < 0,001 post hoc LSD comparisons, p_CS1-CS3=_ < 0,01, p_CS2-CS3=_ < 0,01 and p_CS1-CS2_> 0,05).

**Experiment 4.**

**Implicit aversive memory (fear Pavlovian conditioning).**

*a) Electrodermal Activity (SCR).*During acquisition on Day 1, SCR amplitudes in all groups were significantly higher to the CS1-T than to the rest of the CS (Figure 4, left panel; Mixed repeated measures ANOVA, Stimulus x Group Interaction: F (6,135) = 0,997, p > 0,90, Stimulus x Trial Interaction: F (9,405) = 4,461, p < 0,001, simple effects p_CS1-T vs all CS_ < 0,01) from trial 1 to 5 (p_CS1-T_ < 0,001). On Day 3, memory retention was revealed in CS1-T SCR amplitudes (simple effects p_CS1-T vs all CS_< 0,001)by the absence of difference between the last acquisition trial and the first trial from extinction (p > 0,05). Then, we observed a significant decrease in CS1-T SCR levels from trials 1 – 5 of extinction (simple effects p< 0,01). At the end of extinction training the stimulus did not differ significantly (p_all_> 0,05). Finally the renewal test unveiled the inhibitory process of extinction in all groups, by the significant increase in CS1 SCR levels (*inset,* Two-way ANOVA, Stimulus factor: F (2,135) = 14,40, p < 0,001, post hoc LSD comparisons p_CS1r-CS2r_< 0,001, p_CS1r-CS3r_ p < 0,001 y p_CS2r-CS3r_ > 0,05; Group x Stimulus Interaction: F (4,135) = 0,166, p > 0,05).

*b) US expectancy.*All subjects accurately predicted the US with the CS1-T presentation during acquisition on Day 1 (Figure S2, repeated-measures ANOVA, Context x Trial Interaction: F (9,1098) = 10,430 p < 0,001, Threatening context: Trials 1-2 p_1-2_< 0,001) and expected it during the first trial of extinction on Day 3 (Treatening context p_5-1_< 0,001; simple effects p_contexts_ p < 0,001). Finally, from trials 1-5 of extinction training participants learned that the US was not delivered with any stimulus (Trials 1 -2 p_1-2_< 0,01; Trials 4-5 p_4-5_> 0,05). During renewal test we found the same patterns of results as in previous experiments (Table S1).

*c) Stimuli aversiveness.* Participants evaluation about stimuli aversiveness did not differ across groups (Table S2, Group x Stimulus Interaction: F (4,90) = 0,339, p > 0,05). Fearful CS were rated as more unpleasent than the neutral CS and all stimuli ratings decreased across Day 1 and Day 3 (Trial Factor: F (1,45) = 15,176, < 0,001; Stimulus Factor: F (2,90) = 195,386, p < 0,001, post hoc LSD comparisons: p_CS1-CS3 =_ < 0,01, p_CS2-CS3_< 0,01 and p_CS1-CS2_ > 0,05).

**Supplementary Figures**

**
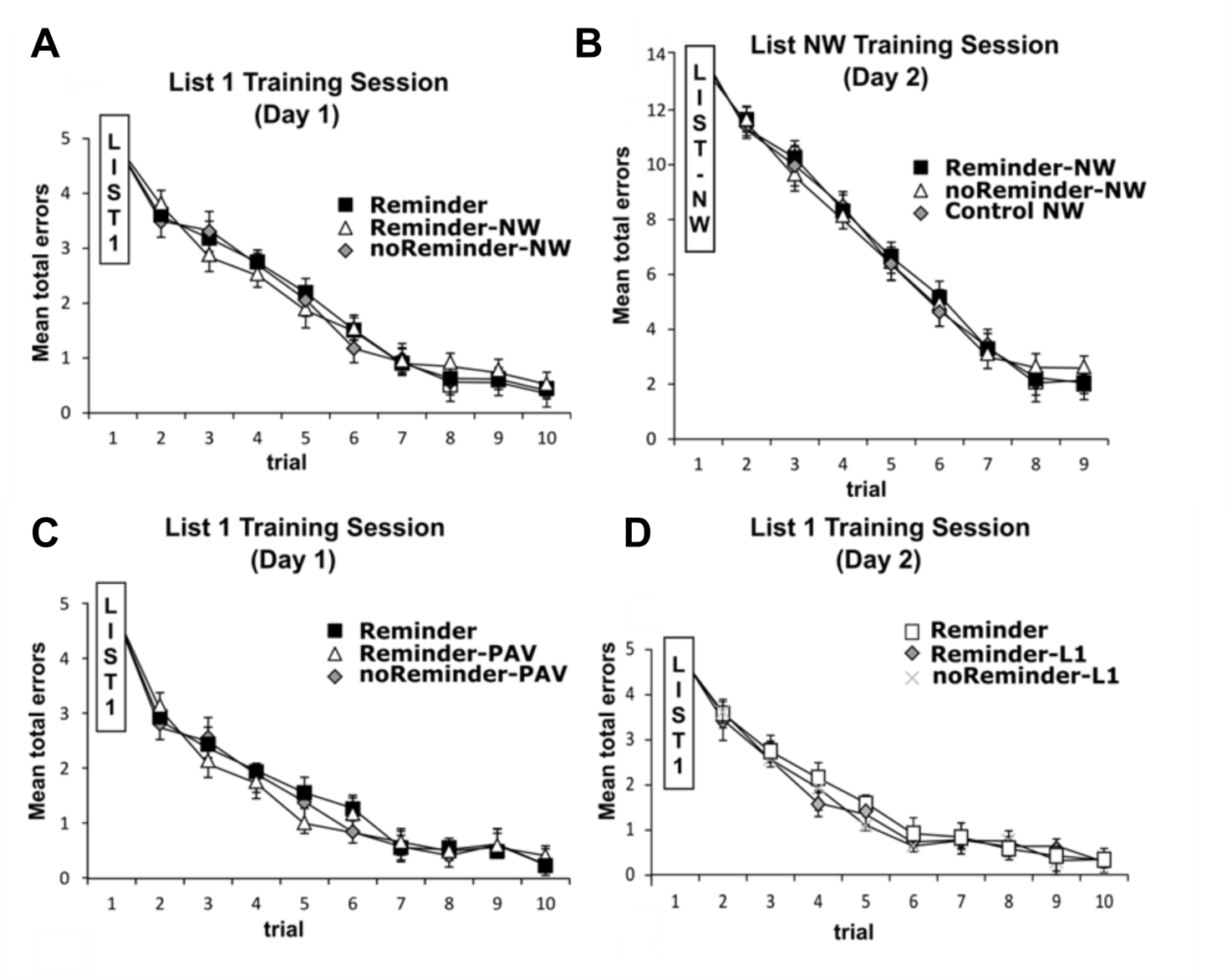
**

**S1 Figure 1. Learning curves**. Mean number of errors +/- SEM per trial on Day 1 or Day 2 for Experiment 1, 3 and 4. On the first trial, the List 1 and the List NW are presented for the first time.


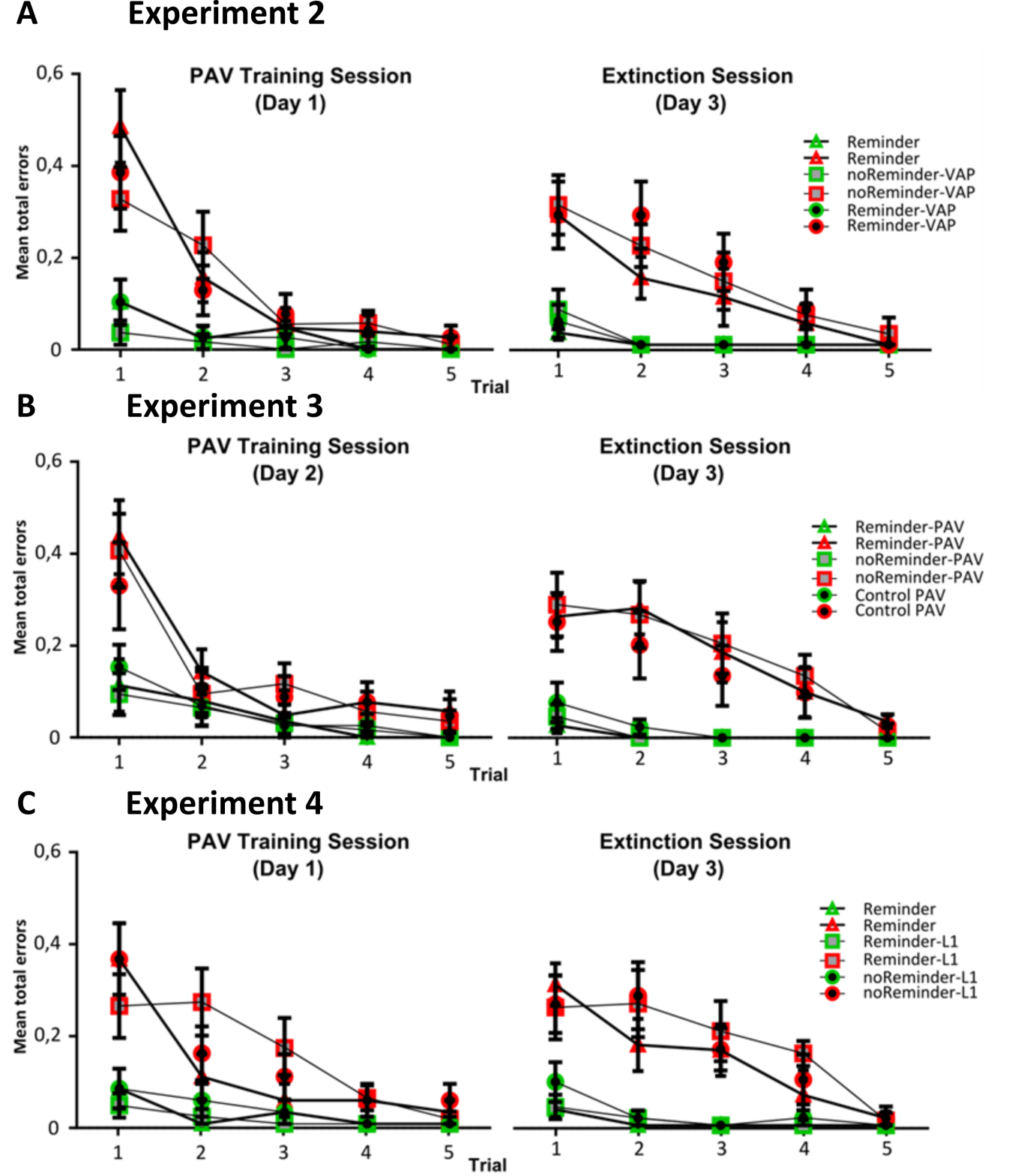


**S2 Figure 2. US expectancy.** Mean total errors +/- SEM in each context per trial on Day 1 (training) and Day 3 (extinction) for Experiments 2, 3 and 4. Red color refers to the threatening context trials (US associated with CS1-T) and green color to the safe context trials (no-US).

**Supplementary Tables**

**TABLE S1- US expectancy: Percentage of YES/NO responses during Renewal test**

| **Experiment 2** | **CS1r** |  | **CS2r** |  | **CS3r** |  |
| --- | --- | --- | --- | --- | --- | --- |
|  | **YES** | **NO** | **YES** | **NO** | **YES** | **NO** |
| **Reminder-PAV** | 51,9 % | 48,1 % | 11,6 % | 88,4 % | 5,8 % | 94,2 % |
| **noReminder-PAV** | 52,8 % | 47,2 % | 7,8 % | 92,2 % | 8 % | 92 % |
| **Control PAV** | 42,5 % | 57,5 % | 5,8 % | 94,2 % | 11,7 % | 88,3 % |
|  |  |  |  |  |  |  |
| **Experiment 3** |  |  |  |  |  |  |
| **Reminder** | 43 % | 57 % | 11,6 % | 88,4 % | 5,8 % | 94,2 % |
| **Reminder-L1** | 52,9 % | 47,1 % | 17,1 % | 82,9% | 8 % | 92 % |
| **noReminder-L1** | 47,2 % | 52,8 % | 5,8 % | 94,2 % | 0% | 100 % |
|  |  |  |  |  |  |  |
| **Experiment 4** |  |  |  |  |  |  |
| **Reminder** | 46,4 % | 53,6 % | 7,8 % | 92,2 % | 7,1 % | 92,9 % |
| **noReminder-VAP** | 51,9 % | 47,2 % | 6,6 % | 93,4 % | 5,8 % | 94,2 % |
| **Reminder-VAP** | 58,2 % | 41,8 % | 11,6 % | 88,4 % | 8,2 | 91,8 % |

**Table S1. US expectancy during Renewal Test.**Percentage of YES/NO responses during the renewal test on Day 3 for CS1r, CS2r and CS3r in Experiments 2, 3 and 4.

|  | **TABLES2 - Mean scores (+/- SEM) for each Stimulus (Aversiveness)** | | | | | | | |
| --- | --- | --- | --- | --- | --- | --- | --- | --- |
| **Experiment 2** | **CS1** |  | **CS2** |  | **CS3** |  | **US** |  |
|  | **TR** | **EXT** | **TR** | **EXT** | **TR** | **EXT** | **TR** | **EXT** |
| **Reminder-PAV** | 7,12 (0,34) | 7,04 (0,28) | 7,01 (0,30) | 6,81 (0,20) | 3,22 (0,28) | 2,87 (0,27) | 7,76 (0,26) | 7,06 (0,44) |
| **noReminder-PAV** | 6,61 (0,27) | 6,50 (0,31) | 7,06 (0,31) | 6,93 (0,24) | 3 (0,30) | 2,75 (0,29) | 7,41 (0,29) | 6,86 (0,49) |
| **Control PAV** | 7,21 (0,36) | 6,68 (0,25) | 7,11 (0,25) | 6,75 (0,26) | 3,12 (0,27) | 2,81 (0,33) | 7,43 (0,28) | 6,75 (0,41) |
|  |  |  |  |  |  |  |  |  |
| **Experiment 3** |  |  |  |  |  |  |  |  |
| **Reminder-L1** | 6,93 (0,34) | 6,43 (0,29) | 7,31 (0,22) | 6,67 (0,31) | 3,43 (0,46) | 3,20 (0,37) | 7,75 (0,35) | 7,12 (0,44) |
| **noReminder-L1** | 6,62 (0,43) | 6,06 (0,32) | 6,95 (0,41) | 6,6 (0,27) | 3,31 (0,36) | 2,81 (0,33) | 7,30 (0,23) | 6,87 (0,30) |
| **Reminder** | 7 (0,23) | 6,87 (0,23) | 6,75 (0,26) | 6,56 (0,25) | 3,12 (0,30) | 2,62 (0,35) | 7,43 (0,25) | 6,93 (0,30) |
|  |  |  |  |  |  |  |  |  |
| **Experiment 4** |  |  |  |  |  |  |  |  |
| **Reminder** | 6,89 (0,28) | 6,52 (0,26) | 7 (0,28) | 6,60 (0,31) | 3,31 (0,35) | 2,90 (0,34) | 7,56 (0,34) | 6,87 (0,40) |
| **noReminder-VAP** | 6,90 (0,27) | 6,45 (0,30) | 6,77 (0,30) | 6,45 (0,26) | 2,95 (0,29) | 2,53 (0,37) | 7,12 (0,28) | 7 (0,41) |
| **Reminder-VAP** | 7,06 (0,24) | 6,61 (0,32) | 6.90 (0,35) | 6,62 (0,31) | 3,06 (0,38) | 2,63 (0,28) | 7,50 (0,29) | 7,10 (0,30) |

**Table S2. Stimulus aversiveness.** Mean scores +/- SEM for each stimulus (CS1, CS2, CS3 and US) at the end of Day 1 (training) and conclusion of Day 3 (Extinction-Renewal) for Experiments 2, 3 and 4. TR refers to the fear Pavlovian conditioning and EXT to extinction training.

|  | **TABLE S3** Mean (±SEM) of the cognitive and physiological measures | | | | |  |  | | | |  |
| --- | --- | --- | --- | --- | --- | --- | --- | --- | --- | --- | --- |
|  | **Experiment 2** | |  |  |  | | |  | |  | |
|  | **Reminder-VAP** | **noReminder-VAP** |  | | | | | |  | | |
|  |  |  |  | | | | | |  | | |
| **SBP (mm HG)** |  |  |  | | | | | |  | | |
| **t0** | 108,06 (1,55) | 107,41 (1,59) | F(3,90)=0,650, p=0,58 | | | | | |  | | |
| **t1** | 102,68 (1,27) | 100,43 (2,30) | SE time VAP _t1-t2_<0,001 | | | | | |  | | |
| **t2** | **114,37 (1,34)** | **115,37 (1,27)** |  | | | | | |  | | |
| **t3** | **111,75 (1,69)** | **110,43 (1,46)** |  | | | | | |  | | |
|  |  |  |  | | | | | |  | | |
| **DBP (mm HG)** |  |  |  | | | | | |  | | |
| **t0** | 76,75 (1,46) | 74,75 (1,85) | F(3,90)=2,14, p=0,100 | | | | | |  | | |
| **t1** | 72,68 (1,04) | 69,26 (1,10) | SE VAP _t1-t2_<0,001 | | | | | |  | | |
| **t2** | **80,01 (1,41)** | **78,75 (1,05)** |  | | | | | |  | | |
| **t3** | **79,50 (1,23)** | **78,43 (1,20)** |  | | | | | |  | | |
|  |  |  |  | | | | | |  | | |
| **HR (LPM)** |  |  |  | | | | | |  | | |
| **t0** | 73,68 (1,94) | 76,51 (1,49) | F(3,90)=1,01, p=0,390 | | | | | |  | | |
| **t1** | 72,12 (2,06) | 74,02 (1,65) | SE VAP _t1-t2_<0,005 | | | | | |  | | |
| **t2** | **79,91 (1,09)** | **79,57 (1,55)** |  | | | | | |  | | |
| **t3** | **78,82 (1,91)** | **78,75 (1,21)** |  | | | | | |  | | |
|  |  |  |  | | | | | |  | | |
| **STAI** |  |  |  | | | | | |  | | |
| **Score** | **-0,96 (0,24)** | **-1,12 (0,31)** | F(1, 30)=0,72 , p=0,51 | | | | | |  | | |
|  |  |  |  | | | | | |  | | |
| **SCL (µS)** |  |  |  | | | | | |  | | |
| **Phase I** | 4,10 (0,28) | 3,91 (0,38) | F(1,31)=0,51 , p=0,480 | | | | | |  | | |
| **Phase III** | **5,55 (0,37)** | **4,98 (0,30)** | SE VAP_I-III_ p<0,001 | | | | | |  | | |
|  |  |  |  | | | | | |  | | |
|  |  |  |  | | | | | |  | | |

**Table S3**. **Social Threatening event (VAP). Cognitive and physiological measures for the Experiment 2.** Cardiovascular activity at different time points (t0, t1, t2, t3). Mean SBP (mm HG), Mean DBP (mm HG) and Mean HR (BPM), Mean Subjective Rating difference and SCL (µS) (+/- SEM) at 4 different time points for the three groups. The F and effect size reported corresponds to the Group x Time interaction of a Repeated Measures ANOVA. SE stands for simple effects.
